# Genotyping-in-Thousands by sequencing of archival fish scales reveals maintenance of genetic variation following a severe demographic contraction in kokanee salmon

**DOI:** 10.1038/s41598-021-01958-0

**Published:** 2021-11-23

**Authors:** Christopher Setzke, Carmen Wong, Michael A. Russello

**Affiliations:** 1 Department of Biology, University of British Columbia, Okanagan Campus, 3247 University Way, Kelowna, BC V1V 1V7 Canada; 2 Parks Canada Yukon Field Unit, Suite 205 - 300 Main St, Whitehorse, YT Y1A 2B5 Canada

**Keywords:** Genetic markers, Conservation genomics, Genetic variation

## Abstract

Historical DNA analysis of archival samples has added new dimensions to population genetic studies, enabling spatiotemporal approaches for reconstructing population history and informing conservation management. Here we tested the efficacy of Genotyping-in-Thousands by sequencing (GT-seq) for collecting targeted single nucleotide polymorphism genotypic data from archival scale samples, and applied this approach to a study of kokanee salmon (*Oncorhynchus nerka*) in Kluane National Park and Reserve (KNPR; Yukon, Canada) that underwent a severe 12-year population decline followed by a rapid rebound. We genotyped archival scales sampled pre-crash and contemporary fin clips collected post-crash, revealing high coverage (> 90% average genotyping across all individuals) and low genotyping error (< 0.01% within-libraries, 0.60% among-libraries) despite the relatively poor quality of recovered DNA. We observed slight decreases in expected heterozygosity, allelic diversity, and effective population size post-crash, but none were significant, suggesting genetic diversity was retained despite the severe demographic contraction. Genotypic data also revealed the genetic distinctiveness of a now extirpated population just outside of KNPR, revealing biodiversity loss at the northern edge of the species distribution. More broadly, we demonstrated GT-seq as a valuable tool for collecting genome-wide data from archival samples to address basic questions in ecology and evolution, and inform applied research in wildlife conservation and fisheries management.

## Introduction

Historical DNA analysis of archival samples has added new dimensions to population genetic studies, expanding our ability to reconstruct patterns and processes of evolution across time and space^1^. Generally defined as preserved hard or soft tissues collected within the past 200 years, data from archival samples have enabled comparisons of genetic diversity and changes in effective population size between past and present populations^1,2^. Furthermore these data can be used to include extirpated populations or extinct species within phylogenetic reconstructions^3–5^.

Calcified material from fish, such as scales, otoliths, and various types of bone, have been collected over the past century and effectively used to investigate individual growth history^6,7^ and response to climate-, population-, and fishing-related pressures^8–11^. In addition, microchemical analyses of these samples have been used to infer fish habitat use^12,13^, origin^14,15^, and exposure to pollutants^16,17^, as well as to reconstruct diet and trophic structure^18–22^. Such material also provides opportunities for recovering historical DNA. For example, mitochondrial DNA obtained from archival scales has been used to determine the genetic effects of historical stocking of Atlantic salmon (*Salmo salar*) in northern Spanish rivers^23^, while microsatellite data from archival and contemporary pectoral fin samples allowed investigation of the temporal genetic consequences of river fragmentation for lake sturgeon (*Acipenser fulvescens*) in Ontario, Canada^24^. More recently, studies have employed single nucleotide polymorphism (SNP) genotyping of archival samples to examine temporal stability of Atlantic cod (*Gadus morhua*) at the northern range margin around Greenland^25,26^ and assess spatiotemporal genetic changes in Atlantic salmon populations across the Baltic Sea^27^. These studies and others have clearly demonstrated the value of historical DNA analysis and temporal population genomics for directly investigating population history and informing fisheries management.

While archival DNA holds great promise for fisheries management, such historical samples can pose challenges as they may contain low DNA quantity, poor DNA quality^28,29^, and a high percentage of exogenous DNA^30^. A range of approaches have been employed for successfully obtaining SNP genotypic data from archival fish samples, including TaqMan® genotyping assays^31^, Illumina GoldenGate assays^25,26^, SNP chips^27^, and shotgun whole genome re-sequencing^32^. One promising approach that has yet to be employed with archival samples is Genotyping-in-Thousands by sequencing (GT-seq)^33^. GT-seq is a multiplex amplicon sequencing method that uses species-specific primer probes to simultaneously target hundreds of loci for up to thousands of individuals, while also reducing amplification of non-target species DNA. Data collection can be conducted using a single library, making preparation simple and cost-effective compared to other methods^34^. Though amplification success can vary during multiplex PCR^35^, GT-seq has been found to amplify consistently with low genotyping error rates^33^. Moreover, GT-seq has been effective in obtaining genotypic data from low quality samples, such as those typically obtained through minimally or non-invasively collected starting materials^36^. Here, we test the effectiveness of GT-seq for genotyping archival samples using as a case study kokanee, the freshwater resident form of sockeye salmon (*Oncorhynchus nerka*), in Kluane National Park and Reserve (KNPR), Yukon, Canada.

**Figure 1:**
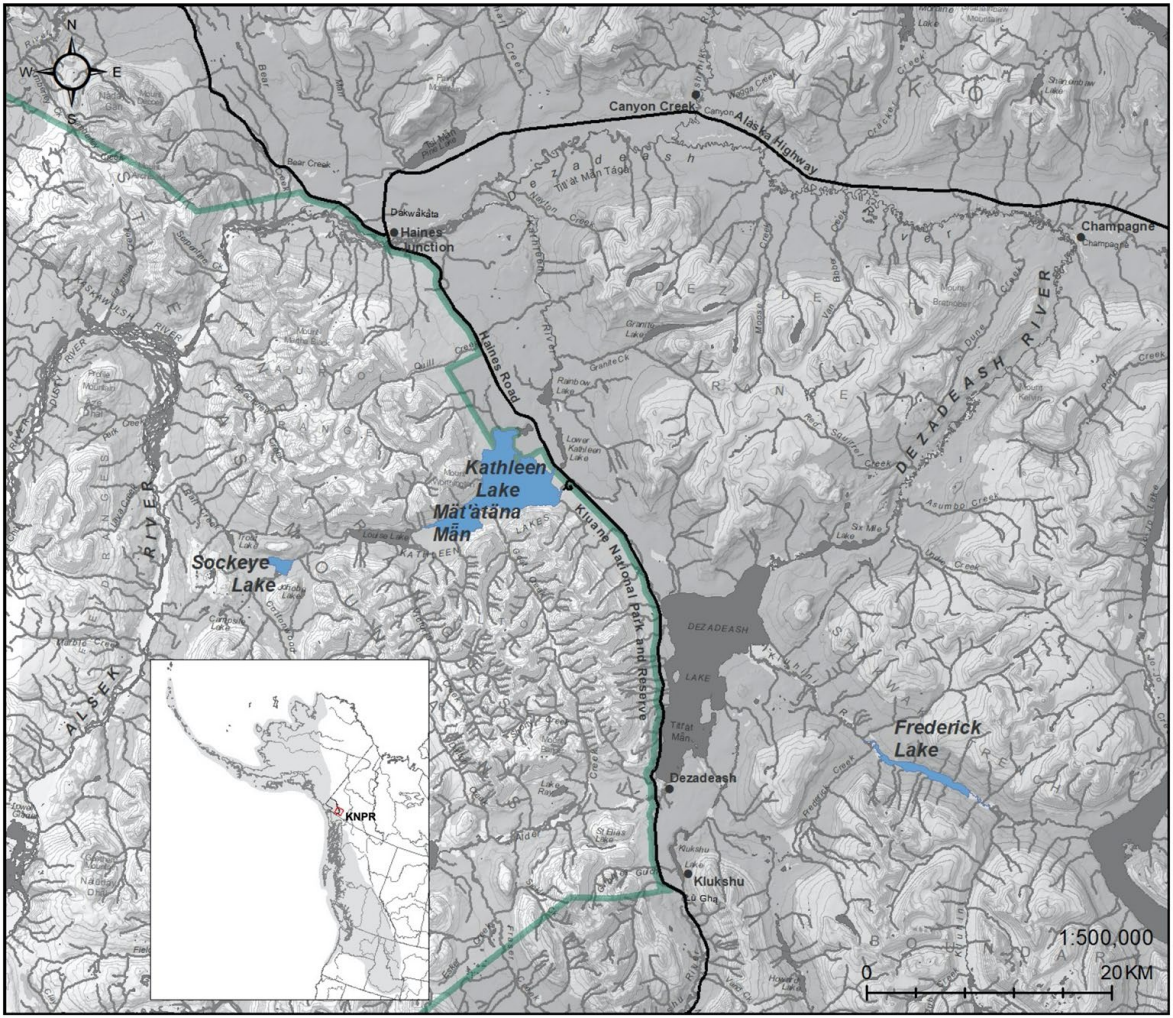
Map of lakes (blue) sampled in Kluane National Park and Reserve (KNPR) (green boundary line) and surrounding area, and the location of KNPR within the North American range of kokanee (inset); map of kokanee distribution (inset) is based on^77^. The base map was modified from^78^.

**Figure 2:**
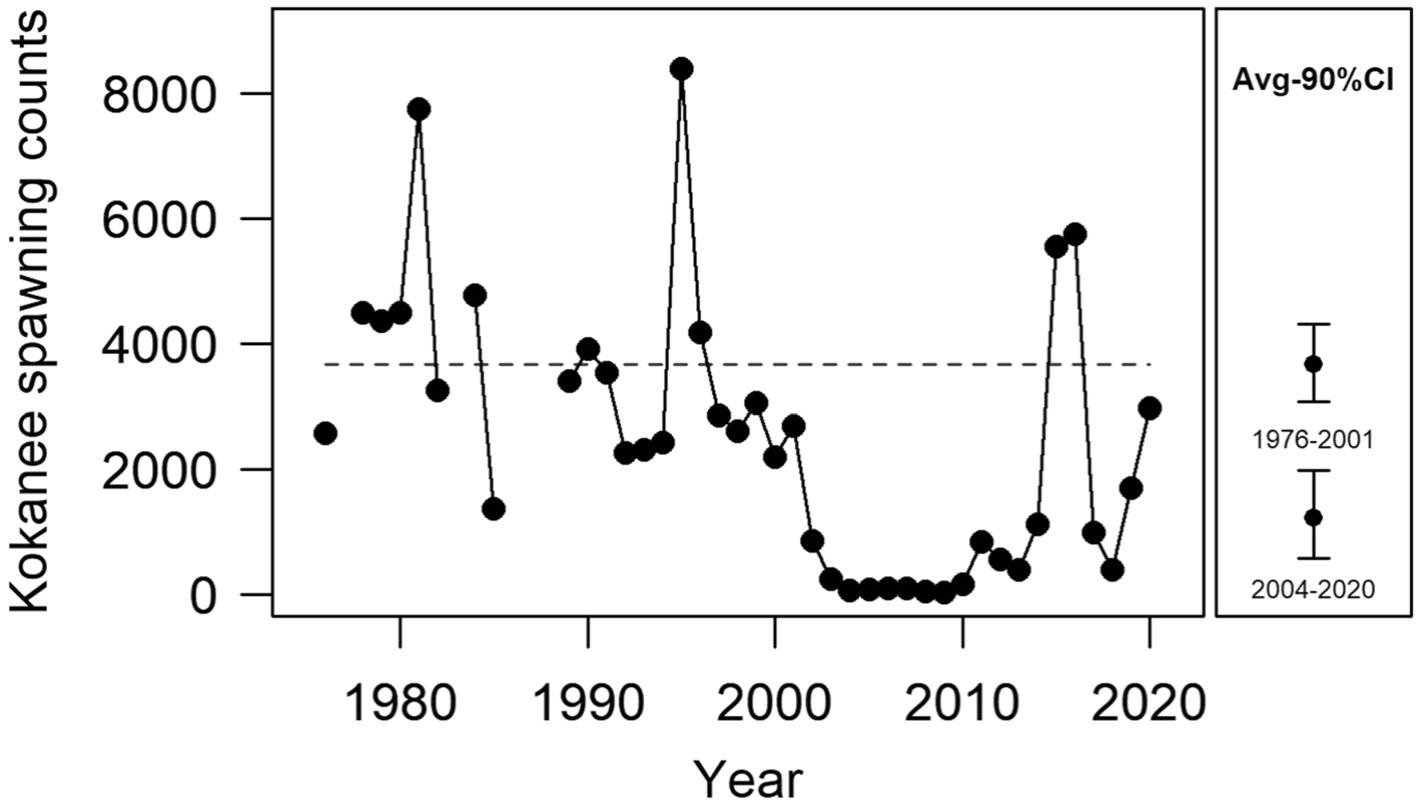
Kokanee spawning counts in Kluane National Park and Reserve, 1973–2020 (Wong, pers. comm) illustrating the annual fluctuations in spawning numbers and severe period of decline from 2002–2014. Data were accessed under the Open Government Licence – Canada (https://open.canada.ca/data/en/dataset/4107dc81-79c0-406e-a1e6-301509d9e506).

Kokanee in and around KNPR represent the northernmost wild populations documented in Canada (Fig. 1), which have experienced large population size fluctuations and extirpations over the past 50 years (Fig. 2). Kokanee within KNPR currently inhabit a connected set of waterbodies including Sockeye, Louise, and Kathleen (Mät’àtäna Män) Lakes (Fig. 1), with spawning in this system primarily occurring in Sockeye Creek and along the north shore of Sockeye Lake. An unconnected population that historically resided just outside of KNPR in Frederick Lake (Fig. 1) is believed to be extirpated since the late 1980’s^37^. Spawning numbers in KNPR have historically averaged ~ 3600 spawners each year, but a severe and prolonged population decline occurred between 2002–2014, reaching a low of just 20 observed spawners in 2009. The population rebounded in 2015 and 2016, with ~ 5550 spawners observed in those years, and has since fluctuated between ~ 400–3000 observed spawners annually (Fig. 2). The genetic consequences of this period of decline and subsequent rebound remain unknown. A recent study investigating the diversity, demographic history, and structure of the contemporary kokanee population in KNPR revealed a pattern of heterozygous excess, a possible signature of a past bottleneck event^38^. Population bottlenecks, in general, can lead to decreased genetic diversity and increased susceptibility to extirpation^39,40^. Genotypic data from archival samples collected pre-crash would allow for the direct investigation of the genetic consequences of population decline in this system.

Here, we employed GT-seq to collect targeted SNP genotypic data from population-level, archival scale samples in Sockeye, Kathleen, and Frederick Lakes collected pre-crash between 1973–1981 and analyzed them relative to data from the contemporary Sockeye Lake post-crash population as well as from a current hatchery stock historically sourced in KNPR. We used the resulting genotypic data to: (1) assess the effectiveness of GT-seq for genotyping archival scales; (2) quantify pre- and post-crash genetic diversity in KNPR; and (3) investigate spatial and temporal population structure in KNPR and Frederick Lake.

## Methods

### Sample collection and DNA extraction

Archival kokanee scale samples from Sockeye Lake were collected July 26–28, 1973 (n = 50) and July 15–August 26, 1975 (n = 13) as a part of a limnological survey of KNPR^41^. Samples from Kathleen Lake were also collected July 13–22, 1973 (n = 22) during the same survey, and during subsequent creel surveys conducted May 26–June 22, 1980 (n = 18), and June 1 – July 23, 1981 (n = 47)^42^. Samples from spawning kokanee in Frederick Lake were collected by angling July 30–31, 1981 (n = 12)^42^. All samples were dried and stored in envelopes. Although carefully labelled and stored, these samples were largely forgotten until unearthed at the KNPR Warden Office, Yukon, Canada, during an office move in 2012 (Wong, pers. comm.). Whole genomic DNA from dry scale samples was extracted following the protocol in^43^ within a dedicated historical DNA laboratory at the University of British Columbia Okanagan.

Previously extracted genomic DNA^38^ from Sockeye Lake and Sockeye Creek kokanee (total n = 46) originally sampled August 25–30, 2019 was used to represent the post-crash population. Also included was previously extracted kokanee DNA from the Whitehorse Rapids Fish Hatchery (n = 29) sampled August 31–September 18, 2019^38^; this population was originally established from eggs and milt collected in Sockeye Creek in 1991–1994, 1999 and 2000 (Wong, pers. comm., LaRocque, pers. comm.). Genomic DNA from two archival and two contemporary samples were subject to Genomic DNA ScreenTape® analysis on an Agilent Tapestation 4150 to assess DNA quality and quantity.

### GT‐seq library preparation and genotyping

GT-seq libraries were constructed following^33^ as modified in^36^. Within library (n = 7 within plates, n = 4 between plates) and between library (n = 3) duplicates were included to allow for estimation of genotyping error rates. Each individual was prepared in two panels using separate sets of previously designed primer pools targeting ~ 100 base pair fragments, one including 288 SNPs^44^ and the other containing 342 SNPs^45^. PCR1 products were diluted 1:20 before use in PCR2. PCR2 products were quantified using PicoGreen™ (Molecular Probes, Inc.), normalized manually to the concentration of the sample with the lowest concentration, and pooled. Pooled samples were purified using MinElute PCR Purification columns (Qiagen) and eluted into 24 μL nuclease-free water. Libraries were sequenced using a Mid Output Reagent Kit (300 cycles) on an Illumina MiniSeq within the Ecological and Conservation Genomics Laboratory at the University of British Columbia Okanagan.

Raw sequence data were genotyped using the GT-seq pipeline (https://github.com/GT-seq/GT-seq-Pipeline). Individuals and SNP loci with > 30% missing data were removed using PLINK v1.90b6.17^46^. Monomorphic loci and those that had been previously identified as outlier loci^44,45^ were removed using VCFTOOLS^47^. Forty loci identified as duplicates between the two panels were also removed.

### Population genetic analyses

Observed (*H*_*o*_) and expected heterozygosity (*H*_*e*_), effective number of alleles (*A*_*e*_), and Weir and Cockerham’s *θ* (999 permutations)^48^ were estimated as implemented in GenoDive^49^. Effective population size (*N*_*e*_) was estimated using the linkage disequilibrium method as implemented in NeEstimator v.2^50^; *N*_*e*_ was only calculated for populations with *n* ≥ 40, as the linkage disequilibrium method provides the most reliable results the closer that sample sizes approximate the true *N*_*e*_^51^. Population structure was visualized using Principal Component Analyses (PCA) as implemented in PCADAPT^52^; the number of principal components (PC) retained was identified using a graphical approach based on the scree plot^53^ as recommended by^52^. The Bayesian clustering approach implemented in STRUCTURE v2.3^54^ was used to infer population structure. Run length was set to 500,000 MCMC (Markov chain Monte Carlo) iterations following a burn-in of 100,000 using correlated allele frequencies under a straight admixture model. STRUCTURE was run with the number of clusters (*K*) varying between 1–9, with 10 replicates for each value of *K*. The most likely *K* was chosen by plotting the log probability (ln Pr(*X|K*)) of the data across the *K* values and choosing the value at which ln Pr(*X|K*) leveled and variance was minimized as recommended in^54^. Bar plots were generated and visualized using CLUMPAK^55^.

## Results

### Sample quality and GT-seq genotypic data processing

Genomic DNA from archival samples were of similar average quantity (archival = 31.5 ng/μL, contemporary = 37.5 ng/μL), but substantially poorer quality, compared to DNA from contemporary samples based on Genomic DNA ScreenTape® analysis (Fig. S1). Average genotyping of loci before filtering was 90.1% across all individuals. After filtering and the removal of outlier loci, monomorphic loci, and duplicate samples, a total of 271 loci were retained across 223 individuals, constituting our neutral dataset. Average read depth was 222.2 reads per loci. Retained individuals included those from Sockeye Lake 1973 (n = 49), Sockeye Lake 1975 (n = 10), Sockeye Lake 2019 (n = 40), Kathleen Lake 1973 (n = 21), Kathleen Lake 1980 (n = 17), Kathleen Lake 1981 (n = 47), Frederick Lake 1981 (n = 11), and the Whitehorse Rapids Fish Hatchery 2019 (n = 28). Genotyping error rates were < 0.01% within GT-seq library (both within and between plates) and 0.60% between GT-seq libraries. A total of 158 loci across 68 contemporary individuals (Sockeye Lake and Hatchery 2019) overlapped between the GT-seq and RADseq data^38^. The genotype discordance between these methods at these loci was 1.77%.

### Population diversity and differentiation

Diversity metrics (*H*_*o*_, *H*_*e*_, *A*_*e*_) from the archival samples were similar and stable across locations and sampling years in Sockeye (1973, 1975) and Kathleen Lakes (1973, 1980, 1981) (Table 1). A slight decrease in *H*_*e*_ and *A*_*e*_ was observed in the Sockeye Lake post-crash population (Table 1). There was a ~ 30% reduction in *N*_*e*_ in Sockeye Lake post-crash [2019: 86.8 (71.4–109.5)] compared to the pre-crash population [1973: 129.0 (104.1–166.8)], although confidence intervals overlapped (Table 1). Differentiation was low between sampling years in Sockeye and Kathleen Lakes, both pre-crash (*θ* ≤ 0.009), and pre-crash compared to post-crash (*θ* ≤ 0.011) (Table 2).

**Table 1:** Diversity estimates including heterozygosity [observed (*H*_*o*_) and expected (*H*_*e*_)], effective number of alleles (*A*_*e*_), and effective population size (*N*_*e*_) with 95% confidence intervals (CIs) for individuals in Sockeye Lake, Kathleen Lake, Frederick Lake, and the Whitehorse Rapids Fish Hatchery (neutral, 271 SNPs).

Sampling location	Year	*n*	*H*_*o*_	*H*_*e*_	*A*_*e*_	*N*_*e*_ (95% CIs)
Sockeye	1973	49	0.252	0.240	1.405	129.0 (104.1–166.8)
Sockeye	1975	10	0.255	0.247	1.403	–
Sockeye	2019	40	0.254	0.230	1.387	86.8 (71.4–109.5)
Kathleen	1973	21	0.250	0.240	1.396	–
Kathleen	1980	17	0.263	0.240	1.398	–
Kathleen	1981	47	0.251	0.237	1.396	127.7 (102.5–167.1)
Frederick	1981	11	0.190	0.179	1.291	–
Hatchery	2019	28	0.229	0.227	1.384	–

Note: *N*_*e*_ was not determined when sample size (*n*) was less than 40.

**Table 2:** Differentiation (Weir and Cockerham’s *θ*)^48^ (above the diagonal) and *p*-values (below the diagonal, bolded values ≤ 0.05) for individuals in Sockeye Lake, Kathleen Lake, Frederick Lake, and the Whitehorse Rapids Fish Hatchery (neutral, 271 SNPs).

	Sockeye 1973	Sockeye 1975	Sockeye 2019	Kathleen 1973	Kathleen 1980	Kathleen 1981	Frederick 1981	Hatchery 2019
Sockeye 1973	–	0.003	0.005	0.000	− 0.003	0.003	0.450	0.033
Sockeye 1975	0.208	–	0.010	0.009	0.003	0.004	0.471	0.035
Sockeye 2019	**0.001**	**0.015**	–	0.011	0.006	0.005	0.465	0.038
Kathleen 1973	0.514	**0.044**	**0.001**	–	− 0.001	0.003	0.462	0.025
Kathleen 1980	0.842	0.237	**0.028**	0.611	–	0.001	0.462	0.038
Kathleen 1981	**0.039**	0.159	**0.001**	0.075	0.310	–	0.453	0.034
Frederick 1981	**0.001**	**0.001**	**0.001**	**0.001**	**0.001**	**0.001**	–	0.476
Hatchery 2019	**0.001**	**0.001**	**0.001**	**0.001**	**0.001**	**0.001**	**0.001**	–

Frederick Lake exhibited substantially lower diversity metrics (Table 1) and significantly higher differentiation (*θ* > 0.450) from Sockeye and Kathleen Lakes (Table 2). Hatchery individuals also had lower observed heterozygosity than detected in both Sockeye and Kathleen Lakes across sampling years, though still higher than Frederick Lake (Table 1). The hatchery population was significantly differentiated from the Frederick Lake population (*θ* = 0.476; Table 2) and exhibited substantially higher *θ* values in comparisons with Sockeye and Kathleen Lakes across all sampling years (Table 2).

**Figure 3:**
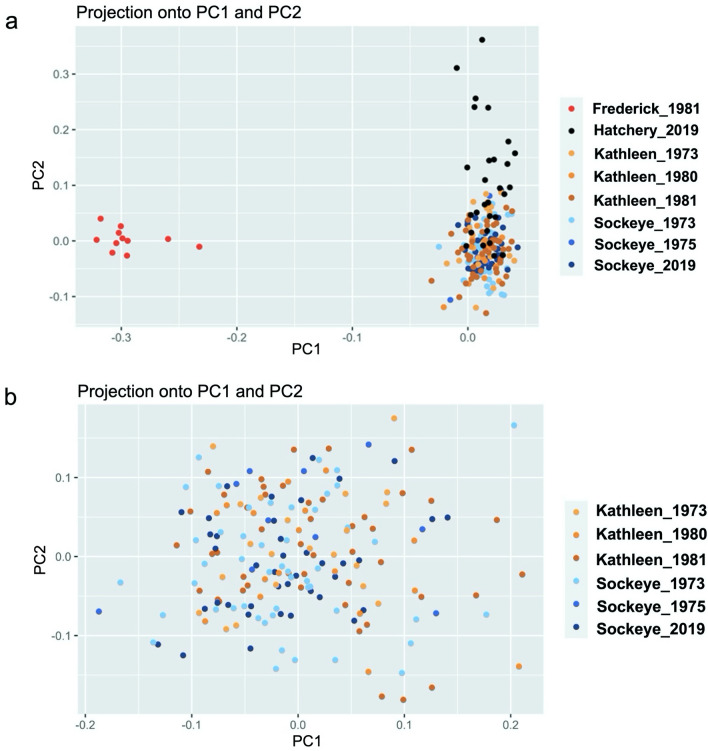
(**a**) PCA for individuals in Sockeye Lake, Kathleen Lake, Frederick Lake, and the Whitehorse Rapids Fish Hatchery (neutral, 271 SNPs) (PC1 = 12.5%, PC2 = 3.7%, genetic variation explained), showing a clear separation of Frederick Lake individuals and a partial separation of hatchery individuals from KNPR individuals. (**b**) PCA for individuals in Sockeye and Kathleen Lakes (neutral, 271 SNPs) (PC1 = 2.9%, PC2 = 2.7%, genetic variation explained) revealing no clear clustering by year or location.

Two PCs were retained from the PCADAPT analysis of all populations across all sampling years; PC1 explained 12.5% of the genetic variation, while PC2 explained 3.7% (Fig. 3a). PC1 separated Frederick Lake from the rest of the individuals, while PC2 separated hatchery individuals from those sampled in Sockeye and Kathleen Lakes. When hatchery and Frederick Lake individuals were removed, there was no clear optimal number of PCs, so two were chosen for the purpose of comparison; both PCs explained very little of the genetic variation (PC1 =  = 2.9%, PC2 = 2.7%). In this reduced analysis, there was no clear clustering by year or location for Sockeye or Kathleen Lake samples (Fig. 3b).

**Figure 4:**
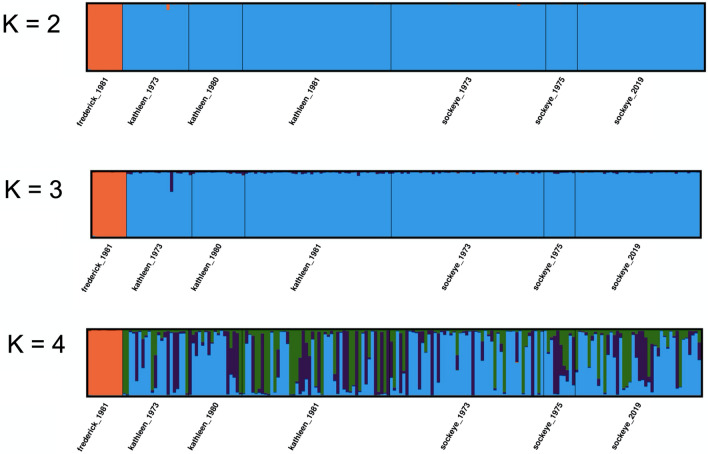
STRUCTURE plots for *K* = 2–4 showing proportion of cluster membership for each individual in Frederick, Kathleen, and Sockeye Lakes (neutral, 271 SNPs). Bayesian clustering analysis of the wild populations implemented in STRUCTURE found evidence for *K* = 2 (Table S1), separating the Frederick Lake population from those sampled in Sockeye and Kathleen Lakes. No structure was found temporally or spatially in or between Sockeye and Kathleen Lakes.

Bayesian clustering analysis of the wild populations implemented in STRUCTURE found evidence for *K* = 2 (Table S1), separating the Frederick Lake population from those sampled in Sockeye and Kathleen Lakes, regardless of sampling year (Fig. 4); additional values of *K* did not reveal further structure within or among Sockeye and Kathleen Lakes, or among sampling years (Fig. 4).

## Discussion

Here we demonstrated GT-seq to be an effective approach for genotyping archival scale samples, exhibiting high coverage (> 90% average genotyping of loci before filtering across all individuals) and low genotyping error (< 0.01% within libraries, 0.60% between libraries) despite the substantially poorer quality of recovered DNA (Fig. S1). These data allowed for the spatial and temporal comparison of diversity metrics and population structure of kokanee in KNPR and surrounding areas, while showing the value of pairing GT-seq and archival DNA analysis more broadly for informing conservation strategies.

### Spatial/temporal population structure and comparison of diversity metrics in KNPR

Overall, we found no evidence of population structure spatially or temporally among the connected lakes where kokanee reside in KNPR (Kathleen and Sockeye), as revealed by extremely low pairwise *θ* values (Table 2) and lack of separation in the PCA or Bayesian clustering analysis (Figs. 3 and 4). The absence of differentiation between Kathleen and Sockeye Lakes is consistent with observations that all kokanee in KNPR, regardless of where they live during other life stages, eventually spawn in the same locations in Sockeye Lake and Creek.

Diversity metrics (*H*_*o*_, *H*_*e*_, *A*_*e*_) were similar temporally and spatially in Sockeye and Kathleen Lakes in pre-crash years, with a slight decrease in *H*_*e*_ and *A*_*e*_ observed in the post-crash population. There was a ~ 30% reduction in *N*_*e*_ in the post-crash population compared to the pre-crash population in Sockeye Lake, although there was some overlap of confidence intervals (Table 1). This finding, as well as the temporal clustering and lack of differentiation between pre- and post-crash populations, suggests that a large proportion of genetic diversity was retained in KNPR kokanee despite the well-documented population crash.

Several factors may affect the genetic outcomes of a demographic contraction, including the severity and duration of the bottleneck, the latter of which can have an outsized effect. Genetic theory predicts that the bulk of genetic diversity can be retained, even during a severe bottleneck, as long as it is not prolonged^56–59^. For example, populations of white-tailed eagles (*Haliaeetus albicilla*) in Europe and Peregrine falcons (*Falco peregrinus*) in North America underwent severe bottlenecks and rapid recoveries as a result of dichlorodiphenyl-trichloroethane (DDT)-containing pesticides and their subsequent bans. However, neither showed a significant loss of genetic diversity as a result of these demographic bottlenecks^60,61^. While these periods of decline lasted decades, the species’ long lifespans likely helped maintain this diversity; in the case of white-tailed eagles, the population bottleneck lasted 20–30 years, which was only equivalent to ~ 2 generations^61^. As kokanee in KNPR have a documented generation time of ~ 4–5 years^42^, it is likely the demographic contraction in this system only lasted ~ 2–3 generations. Though severe, this duration of the bottleneck may not have been long enough to significantly erode genetic diversity within the population.

### Extirpated Frederick Lake

Our results revealed the extirpated Frederick Lake population to be genetically distinct from kokanee in KNPR, as evidenced by high levels of differentiation (*θ* > 0.450; Table 2) and patterns of clustering in the PCA and STRUCTURE analyses (Figs. 3A, 4). The two competing hypotheses of the origin of kokanee in KNPR and Frederick Lake have assumed that they originated from the same event, either by way of colonization from the (1) Alsek River or (2) Tatshenshini River to Klukshu Lake (Łu Ghą Män)^42^. Yet, the high differentiation between these populations may suggest that they originated from separate ancestral populations over multiple events. Further research that includes sampling of anadromous sockeye salmon populations in the Alsek River and Klushu Lake would be required to test the single/multiple origin hypotheses. Regardless, the extirpation of Frederick Lake kokanee represents a loss of biodiversity, which may be even more pronounced given its northerly distribution, as small, isolated populations at the periphery of a species range,  may harbor unique traits not found in the core of a species range^62,63^.

Though it is difficult to conclude what led to the extirpation of the Frederick Lake population in the late 1980’s^37^, the population exhibited much lower diversity metrics in terms of *H*_*o*_, *H*_*e*_, and *A*_*e*_ than the population in KNPR, as well as the Whitehorse Rapids Fish Hatchery population (Table 1). There is general agreement that genetic diversity is vital to a population’s viability^39,64^. As a lack of diversity has been associated with the extinction or extirpation of many species or populations^65,66^, the low levels of genetic diversity seen in Frederick Lake kokanee may have contributed to the loss of this population; however, additional studies would be required to test this hypothesis.

### Value of archival samples in conservation

Our study highlights the valuable insights archival samples can provide to fisheries conservation and management. For example, previous RADseq data showed the contemporary population in KNPR displayed a heterozygous excess^38^, which is one potential indicator of a genetic bottleneck^67^. Yet, genotypic data from archival samples in KNPR revealed that this heterozygous excess was likely a feature of the population, rather than a result of a recent bottleneck. If only contemporary data were available, conservation strategies, such as initiatives to propagate diversity through hatchery supplementation, may have been implemented, possibly in vain or with negative consequences^38^. That said, we did observe a slight decrease in some diversity metrics over time in KNPR, such as *H*_*e*_ and *A*_*e*_, as well as a small, but not significant, reduction in *N*_*e*_ post-crash; these findings warrant continued monitoring to document trends, examine outcomes, and inform conservation planning moving forward.

While potential archival samples such as fish scales and otoliths are generally collected during surveys or studies, this material is sometimes only used to meet short-term objectives, with long-term preservation and storage given little priority. Although such samples are well preserved and recorded at some institutions, in others, standard operating procedures include retaining only the most recent five years of samples due to insufficient storage space^68^. Even where sufficient storage space exists, maintenance of archival material may be inconsistent or undervalued, leading to variable quality of samples^69^. In our case, the last remaining physical evidence of an assumed extirpated population was at risk of being discarded during an office move 40 years after collection. As the use of genomics in fisheries conservation and management continues to progress^70,71^, proper long-term storage and maintenance of archival samples, potentially through centralized storage facilities and collection management systems^72,73^, will be key in preserving their value for future studies.

### Utility of GT-seq in studies using archival samples

Our study shows how GT-seq can be used to effectively and efficiently genotype degraded archival samples. Though diversity metrics in the historical GT-seq dataset were slightly lower than what was found using RADseq in the same contemporary individuals, similar trends, such as heterozygote excess, were observed in both^38^. These lower diversity metrics were most likely due to the inclusion of unique Frederick Lake individuals while calling SNPs rather than GT-seq itself. As a case in point, when Frederick Lake individuals were removed from the dataset, diversity metrics between the same individuals were similar between the RADseq and GT-seq datasets (Table 3).

**Table 3:** Comparison of neutral RADseq^38^ and GT-seq dataset diversity estimates including heterozygosity [observed (*H*_*o*_) and expected (*H*_*e*_)] and effective number of alleles (*A*_*e*_).

Sampling location	Method	*n*	# of SNPs	*H*_*o*_	*H*_*e*_	*A*_*e*_
Sockeye_2019	RADseq	40	11,442	0.300	0.292	1.458
Hatchery_2019	RADseq	28	11,442	0.264	0.274	1.429
Sockeye_2019	GT-seq	40	229	0.301	0.273	1.458
Hatchery_2019	GT-seq	28	229	0.271	0.269	1.455

While a range of methods have been used for genotyping archival DNA^25–27,31,32^, our study is the first to our knowledge to employ GT-seq, which has many advantages compared to other approaches. First, GT-seq only requires amplicon lengths of ~ 100 base pairs for effective genotyping^33^, positioning it as a suitable approach to apply to highly fragmented DNA that can be commonly obtained from archival samples of varying ages.

Second, GT-seq has a relatively low genotyping error rate^33^. High genotyping error is often a serious problem for historical samples, mainly due to nucleotide misincorporation during the amplification of archival DNA^1,29^. Estimates of genotyping error using more traditional methods, such as microsatellite fragment analysis, can range as high as 17–21%^1,74,75^. In our study there was a < 0.01% genotyping error rates within libraries and 0.60% error rate between libraries, while average genotyping of loci before filtering was 90.1% across all individuals, highlighting the ability of GT-seq to obtain high quality data with minimal error.

Third, GT-seq makes use of a straightforward protocol that is relatively cost-effective, allowing for the simultaneous genotyping of hundreds or thousands of individuals at hundreds of loci. Though panel design requires upfront development and investment, once optimized, multi-locus genotypes can be obtained for ~ $6.00 (USD) per sample^34^. When compared to RADseq, which costs ~ $30.00 per sample, or a targeted capture sequencing approach such as Rapture, which costs ~ $15.00 per sample, GT-seq is extremely cost-effective when processing hundreds or thousands of samples^34^. Moreover, GT-seq does not require highly specialized equipment for library preparation and uses simple scripts for bioinformatic processing of the recovered sequence data^33,34^. This reduces barriers to entry for research groups without advanced instrumentation or strong computational backgrounds to use this approach, while also standardizing methods across labs^34^.

Lastly, GT-seq can provide connectible data to monitor populations over time. Once designed, GT-seq panels can be employed by any lab or facility to directly target specific SNPs at specific locations in the genome. This is unlike more traditional markers, such as microsatellites, which are indirectly assayed using fragment analysis^76^. As such, GT-seq panels can be used not only as a means to compare genetic diversity and detect changes in effective population size and structure between populations past and present, but also to continually monitor these parameters into the future.

Taken together, these advantages make GT-seq a valuable tool for implementing genomics into conservation^34^. This approach has been previously shown to be effective for low quality DNA from minimally and non-invasively collected samples^36^. Here, we have demonstrated the utility of GT-seq for genotyping archival samples, further extending the temporal and spatial resolution this method can bring for addressing basic questions in ecology and evolution, as well as for informing applied research in wildlife conservation and fisheries management.

## Supplementary Information

Supplementary Information.

## Data Availability

All Illumina raw reads are available from the NCBI sequence read archive (BioProject ID: PRJNA769146). SNP genotypic data are deposited in DRYAD (10.5061/dryad.qfttdz0j2).
