# Supplementary material for: Genotyping-in-Thousands by sequencing of archival fish scales reveals maintenance of genetic variation following a severe demographic contraction in kokanee salmon
Source: Sci Rep. 2021 Nov 23;11:22798. doi: 10.1038/s41598-021-01958-0 (PMC8611073; doi:10.1038/s41598-021-01958-0)
Supplement: Supplementary file 1 — Supplementary Information. [file 41598_2021_1958_MOESM1_ESM.docx]

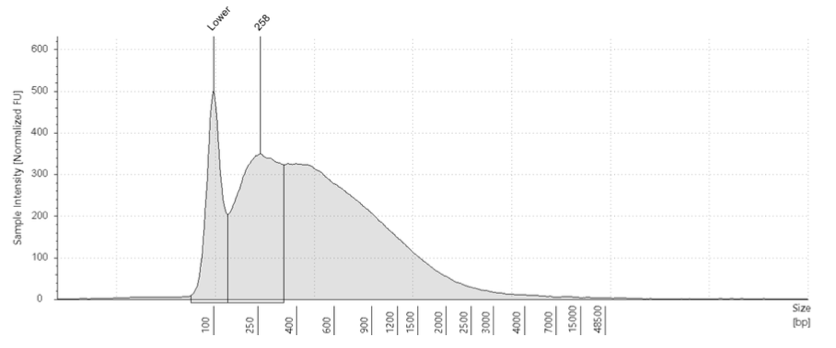

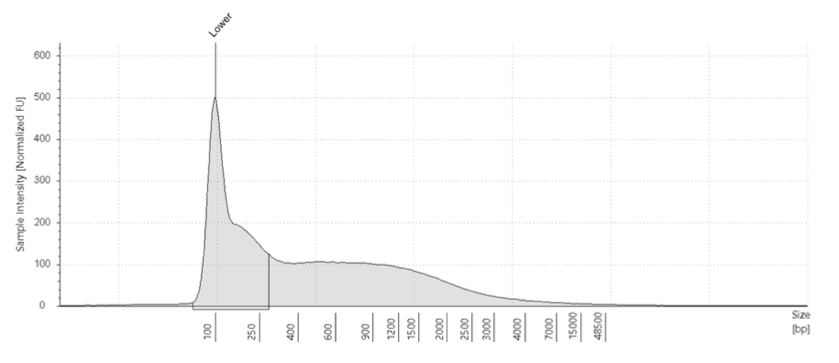


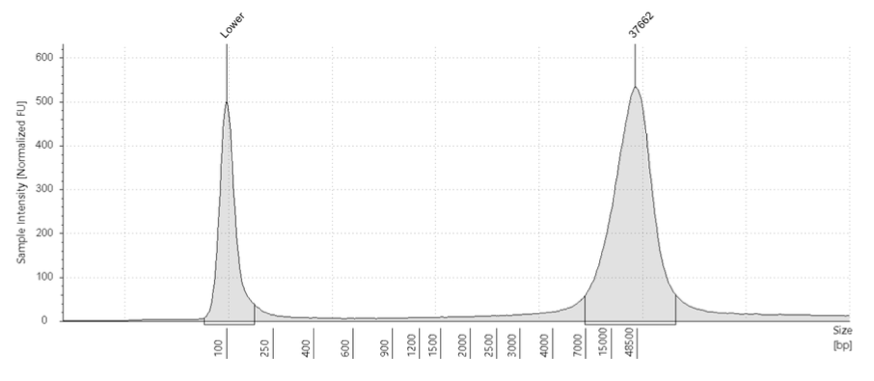


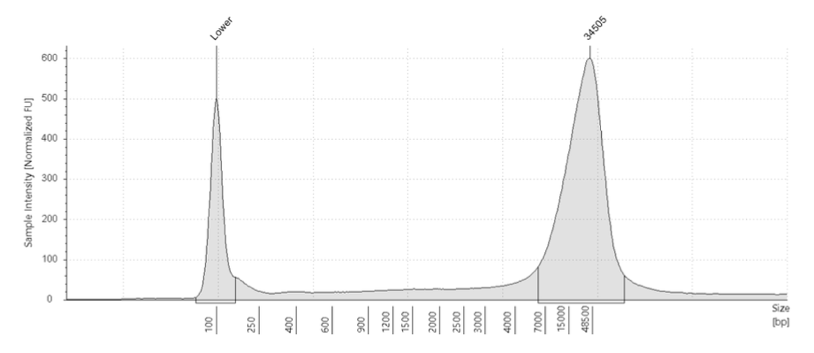


Figure S1: Base pair sample intensity of DNA from archival dry scale (top) and contemporary fin clip (bottom) samples from Genomic DNA ScreenTape®.

Table S1: Summarized STRUCTURE output of posterior probabilities and variance for *K* = 1-9.

| ***K*** | **Reps** | **Mean LnP(K)** | **Stdev LnP(*K*)** | **Ln’(*K*)** | **\|Ln’’(*K*)\|** | **Delta *K*** |
| --- | --- | --- | --- | --- | --- | --- |
| 1 | 10 | -39608.60 | 0.14 | — | — | — |
| 2 | 10 | -36273.36 | 0.20 | 3335.24 | 4334.23 | 21551.75 |
| 3 | 10 | -37272.35 | 2002.34 | -998.99 | 2251.00 | 1.12 |
| 4 | 10 | -36020.34 | 2.02 | 1252.01 | 1245.50 | 616.41 |
| 5 | 10 | -36013.83 | 15.79 | 6.51 | 2.98 | 0.19 |
| 6 | 10 | -36004.34 | 9.73 | 9.49 | 79.33 | 8.15 |
| 7 | 10 | -36074.18 | 17.70 | -69.84 | 336.57 | 19.01 |
| 8 | 10 | -36480.59 | 404.63 | -406.41 | 223.04 | 0.55 |
| 9 | 10 | -36663.96 | 212.78 | -183.37 | — | — |
